# Culture media profoundly affect *Candida albicans* and *Candida tropicalis* growth, adhesion and biofilm development

**DOI:** 10.1590/0074-02760160294

**Published:** 2016-10-03

**Authors:** Manjula M Weerasekera, Gayan K Wijesinghe, Thilini A Jayarathna, Chinthika P Gunasekara, Neluka Fernando, Nilwala Kottegoda, Lakshman P Samaranayake

**Affiliations:** 1 University of Sri Jayewardenepura, Faculty of Medical Sciences, Department of Microbiology, Nugegoda, Gangodawila, Sri Lanka; 2 University of Sri Jayewardenepura, Faculty of Applied Sciences, Department of Chemistry, Nugegoda, Sri Lanka; 3 Sri Lanka Institute of Nanotechnology, Nanoscience and Technology Park, Pitipana, Homagama, Sri Lanka; 4 University of Sri Jayewardenepura, Center for Advanced Material Research, Nugegoda, Sri Lanka; 5 University of Queensland, Department of Oral Microbiomics and Infection, Brisbane, Australia

**Keywords:** *Candida* biofilms, culture media, SDB, YNB, RPMI 1640

## Abstract

As there are sparse data on the impact of growth media on the phenomenon of biofilm development for *Candida* we evaluated the efficacy of three culture media on growth, adhesion and biofilm formation of two pathogenic yeasts, *Candida albicans* and *Candida tropicalis*. The planktonic phase yeast growth, either as monocultures or mixed cultures, in sabouraud dextrose broth (SDB), yeast nitrogen base (YNB), and RPMI 1640 was compared, and adhesion as well as biofilm formation were monitored using MTT and crystal violet (CV) assays and scanning electron microscopy. Planktonic cells of *C. albicans*, *C. tropicalis* and their 1:1 co-culture showed maximal growth in SDB. *C. albicans/C. tropicalis* adhesion was significantly facilitated in RPMI 1640 although the YNB elicited the maximum growth for *C. tropicalis*. Similarly, the biofilm growth was uniformly higher for both species in RPMI 1640, and *C. tropicalis* was the slower biofilm former in all three media. Scanning electron microscopy images tended to confirm the results of MTT and CV assay. Taken together, our data indicate that researchers should pay heed to the choice of laboratory culture media when comparing relative planktonic/biofilm growth of *Candida*. There is also a need for standardisation of biofilm development media so as to facilitate cross comparisons between laboratories.

*Candida* species are commensals in about 75% of healthy individuals (Ghannoum et al. 2010) and cause opportunistic infections in compromised patient groups (Scully et al. 1994). Amongst the pathogenic *Candida* species *C. albicans* is generally considered the most virulent and the commonest fungal pathogen in humans (Cannon et al. 1995) followed by *C. tropicalis* (Cannon et al. 1995). Both species have the ability to form biofilms and are responsible for a whole array of biofilm-related infections (Douglas 2002).

Biofilms are defined as microbial communities attached to either a biotic or an abiotic surface and encased in an extracellular matrix (Ramage et al. 2001). It has been estimated that up to 65% of human infections on implanted biomaterials and host surfaces are due to formation of biofilms (Pierce et al. 2008). The biofilm mode has unique characteristics in contrast to the planktonic form, such as their exquisite resistance to antimicrobials (Ramage et al. 2001, Zijnge et al. 2010) mainly due to the extracellular matrix encasing the biofilm. In comparison to the biofilm architecture of *C. albicans*, comprising yeast blastospores, hyphae and pseudohyphae, *C. tropicalis* biofilms exist essentially as a multilayer structure of yeast blastospores; yet in both cases, the biofilms are embedded in extracellular matrix (Al-Fattani & Douglas 2006). Despite their structural diversity, biofilm development of both species are known to be dictated by the quality of the substrate biomaterial, the growth medium (Fracchia et al. 2010), carbohydrate source and concentration (Jin et al. 2004) and pH (Marsh 2006).

Historically, biofilms of *Candida* have been evaluated in laboratory settings using a model system such as microtiter plates (Nett et al. 2011), flow cells (Foster & Kolenbrander 2004), constant depth film fermenters (Douglas 2002), an artificial mouth model system (Rasiah et al. 2005, Weerasekera et al. 2013) and perfused biofilm fermenters (Douglas 2002). Of these, the microtiter plate system is the most popular due to its versatility, simplicity, reproducibility, and efficacy. Many researchers have studied *Candida* biofilms using this system particularly the biofilm architecture either in monocultures or mixed cultures (Jin et al. 2004, Bandara et al. 2010, Nett et al. 2011).

Despite burgeoning data of the effect of varying culture media on candidal growth (Serrano-Fujarte et al. 2015), adhesion and biofilm development in laboratory settings there is no consensus recommendation to date, for a specific, choice medium suitable for in vitro biofilm experiments. Therefore, we investigated the effect of three different culture media on the growth, adhesion and biofilm formation of *C. albicans* and *C. tropicalis*, and their mixed biofilms using the microtiter plate system.

## MATERIALS AND METHODS

*Strains and culture conditions* - *C. albicans* (ATCC 10231) and *C. tropicalis* (ATCC 13803) type strains were used in this study. Cultures were maintained on Sabouraud Dextrose Agar (SDA, Sigma-Aldrich, USA) slants in stock cultures. Stock cultures were subcultured onto freshly prepared SDA plates and incubated at 35ºC for 48 hours. For all planktonic and biofilm assays, Yeast Nitrogen Base (YNB, Sigma-Aldrich, USA) supplemented with 100 mM glucose, Sabouraud Dextrose Broth (SDB, HiMedia, India) and RPMI 1640 (Gibco, USA) were used as culture media.

*Planktonic growth assay* - Planktonic growth rate was determined as described previously (Jin et al. 2004) with modifications. Briefly, 10^6^ cells/mL suspensions of *C. albicans* and *C. tropicalis* were prepared in sterile YNB supplemented with 100 mM glucose, SDB and RPMI 1640. A 1:1 suspension of *C. albicans* and *C. tropicalis* cells was also prepared. A volume of 100 µL of organism suspensions were inoculated in triplicate into a sterile, flat bottom, polystyrene 96 wells microtiter plate. The growth rate of planktonic *Candida* cells was determined by optical density measurement of the suspensions in each well at 492 nm (Jin et al. 2004) at 2 h intervals for 14 h using a microtiter plate reader (SPECTRAmaxPLUS384 Molecular Devices, Inc, USA), and growth curves were prepared.

*Candida adhesion assay* - Standard cell suspensions (10^7^ cells/mL) of *C. albicans* and *C. tropicalis* were prepared in sterile YNB containing 100 mM glucose, SDB and RPMI 1640. In addition to the monospecies suspensions, 1:1 mixture of a dual species suspensions were prepared by mixing equal volumes of each species suspension.

First, 100 µL/well standard cell suspensions of *C. albicans*, *C. tropicalis* and mixed species were inoculated in triplicate in to wells of a sterile flat bottomed microtiter plate and incubated for 90 min at 37ºC for initial adhesion (Jin et al. 2004). After 90 min incubation, the plate was washed carefully twice with 200 µL of sterile phosphate buffered saline (PBS) and the adherent cells were quantified using crystal violet (CV) (HiMedia, India) assay (Traba & Liang 2011) and MTT (Traba & Liang 2011, Tsang et al. 2012) (tetrazolium salt 3-[4, 5-dimethylthiazol-2-yl]-2, 5-diphenyltetrazolium bromide, (Sigma-Aldrich, USA) assay with modifications.

For CV assay, 100 µL of 1% CV solution was added to each well and incubated for 20 min at 37ºC and plate was washed carefully thrice with sterile PBS. Finally CV stained cells were decolorised by adding 200 µL/well of 95% ethanol. Then 100 µL of ethanol was transferred to a new microtiter plate and absorbance was measured at 595 nm using a microtiter plate reader (SPECTRAmaxPLUS384 Molecular Devices, Inc, USA). For MTT assay 1 mg/mL working solution of MTT was prepared by diluting 5 mg/mL stock solution. To quantify the adhered living cell mass, 50 µL of working solution was added to each well and incubated at 37ºC for 4 h. After incubation, MTT solution was carefully aspirated. Dimethyl sulfoxide (Sigma-Aldrich, USA) 100 mL was added to each well to solubilize the formazon product and absorbance was measured by setting the detection and reference wavelengths at 570 nm and 630 nm, respectively.

*Biofilm development assay* - Growth rate assay was performed using the method described previously (Jin et al. 2004) with modifications. Briefly, standard cell suspensions of *C. albicans*, *C. tropicalis* and mixed species were prepared in sterile PBS. Standard cell suspensions (100 µL) were inoculated in triplicate to sterile flat bottomed microtiter plates and the plates were incubated for 90 min at 37ºC for initial adhesion. After the adhesion phase, the plates were washed twice with 200 µL/well sterile PBS to remove non-adherent cells and the wells were filled with 100 µL/well sterile culture media. Plates were incubated at 37ºC up to 96 h and the culture medium were replenished daily. The biofilm cell mass was quantified at 24, 48, 72 and 96 h using CV assay and MTT assay as described above. Growth curves were prepared in order to determine the effect of the three different media on biofilm growth.

*Scanning electron microscopy (SEM)* - For examination of ultrastructure, biofilms of *C. albicans* were formed on 10 mm diameter coverslips, processed and examined. Coverslips were pretreated with concentrated sulphuric acid, 95% ethanol and foetal bovine serum prior to biofilm formation (Pu et al. 2014). Formed biofilms were fixed with 2.5% glutraldehyde for 2 h and serially dehydrated with ethanol. After overnight drying in a desiccator, biofilms were coated with gold and examined using the SEM (Hitachi SU 6600) (Harriott & Noverr 2009, Tsang et al. 2012).

*Statistics* - Each experiment was performed in triplicates on two different occasions. The statistical analyses were performed using SPSS16.0 for windows. One-way ANOVA and two-way ANOVA were performed to analyse the differences among multiple means. P-values of < 0.05 were considered statistically significant.

## RESULTS

**Fig. 1:**
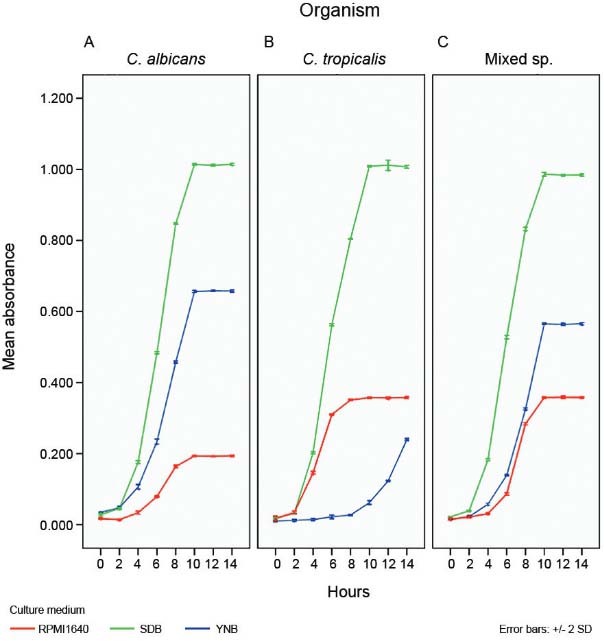
: growth curves of planktonic *Candida* in three different culture media: yeast nitrogen base (YNB), sabouraud dextrose broth (SDB) and RPMI 1640. *C. albicans* (A), *C. tropicalis* (B) and 1:1 mixed species (C) growth in YNB, SDB and RPMI. Data are presented as mean (± standard deviation) of three independent experiments performed in triplicates.

*Growth rate of planktonic Candida in different culture media* - In the YNB medium supplemented with 100 mM glucose, both *C. albicans* and the 1:1 mixed species co-culture reached a plateau at 10 h (Fig. 1A). The maximum growth was observed in *C. albicans* mono culture followed by the 1:1 mixed species co-culture and *C. tropicalis*. In both SDB and RPMI 1640 medium, all three cultures reached a plateau at 10 h (Fig. 1B-C). However the growth of *C. albicans* was lower than the *C. tropicalis* and 1:1 mixed species co-culture in RPMI 1640 medium (Fig. 1C).

On comparing the relative growth of the yeasts in culture media the maximum growth of all three planktonic cultures was achieved with SDB (Fig. 1A-C).

**Fig. 2:**
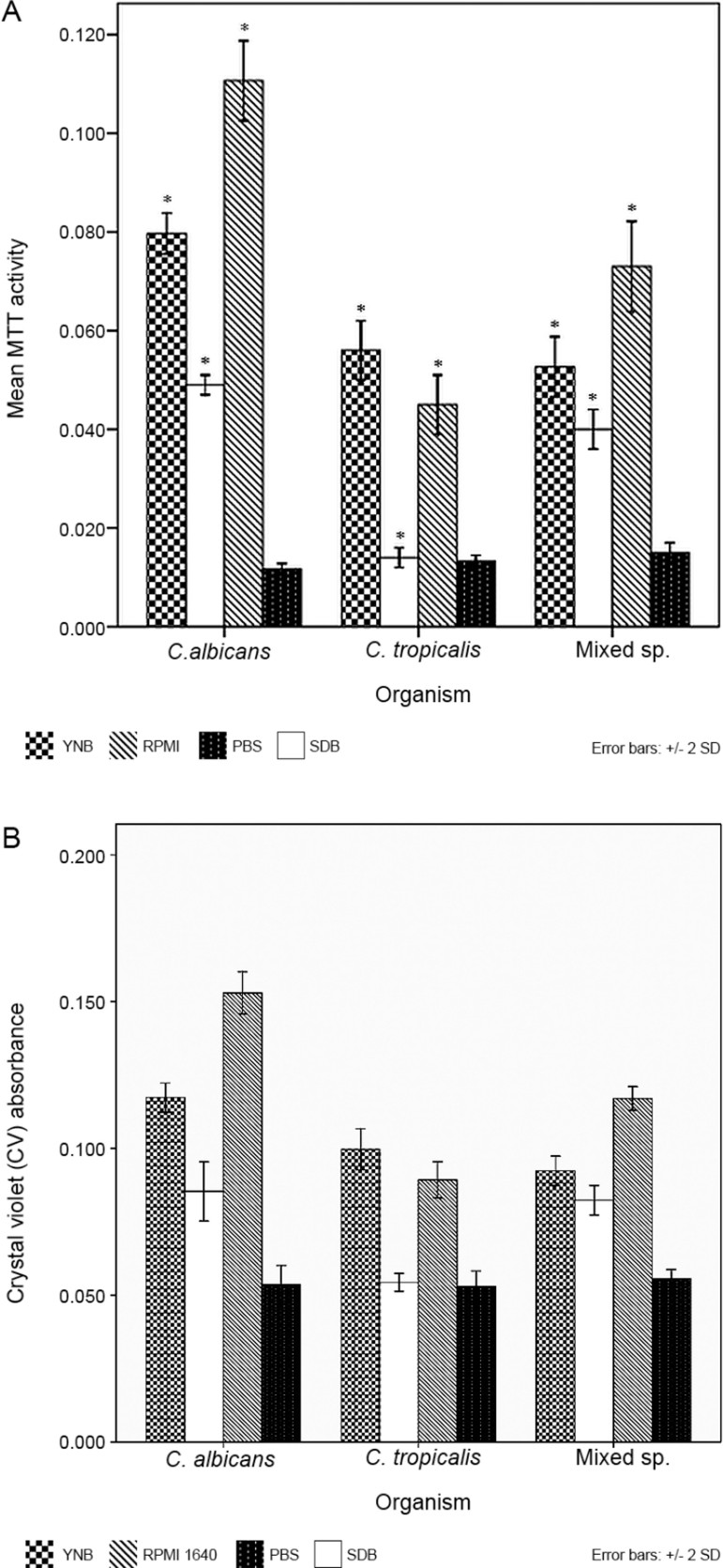
: adhesion of *Candida albicans*, *C. tropicalis* and 1:1 mixed species in yeast nitrogen base (YNB), sabouraud dextrose broth (SDB) and RPMI 1640 media. (A) MTT assay (B) crystal violet (CV) assay. Adhesion in phosphate buffered saline (PBS) served as the control. All error bars represent the standard deviations (SD). For comparison of relative adhesion of *Candida* species in different culture media, a two-way ANOVA was performed followed by a Bonferroni post hoc test. *: indicates a significant difference in adhesion between three culture media for given organism, with a p < 0.05 using ANOVA.

*Effect of culture media on yeast adhesion* - Both CV and MTT assays were carried out to evaluate the adhesion of *Candida* species. And the results correlated well. *C. albicans* and 1:1 mixed species co-cultures demonstrated increased adhesion in all three culture media compared with the PBS control (p < 0.001) (Fig. 2A-B). *C. tropicalis* cells showed the maximal adhesion in YNB supplemented with 100 mM glucose but SDB had no significant effect on adhesion compared to the PBS control (p = 0.981). Maximum adhesion was noted with RPMI 1640 for both *C. albicans* and the 1:1 mixed species co-cultures (Fig. 2A-B). There was no difference in adhesion after 90 min when PBS was used as a negative control for *C. albicans*, *C. tropicalis* and 1:1 mixed species.

**Fig. 3:**
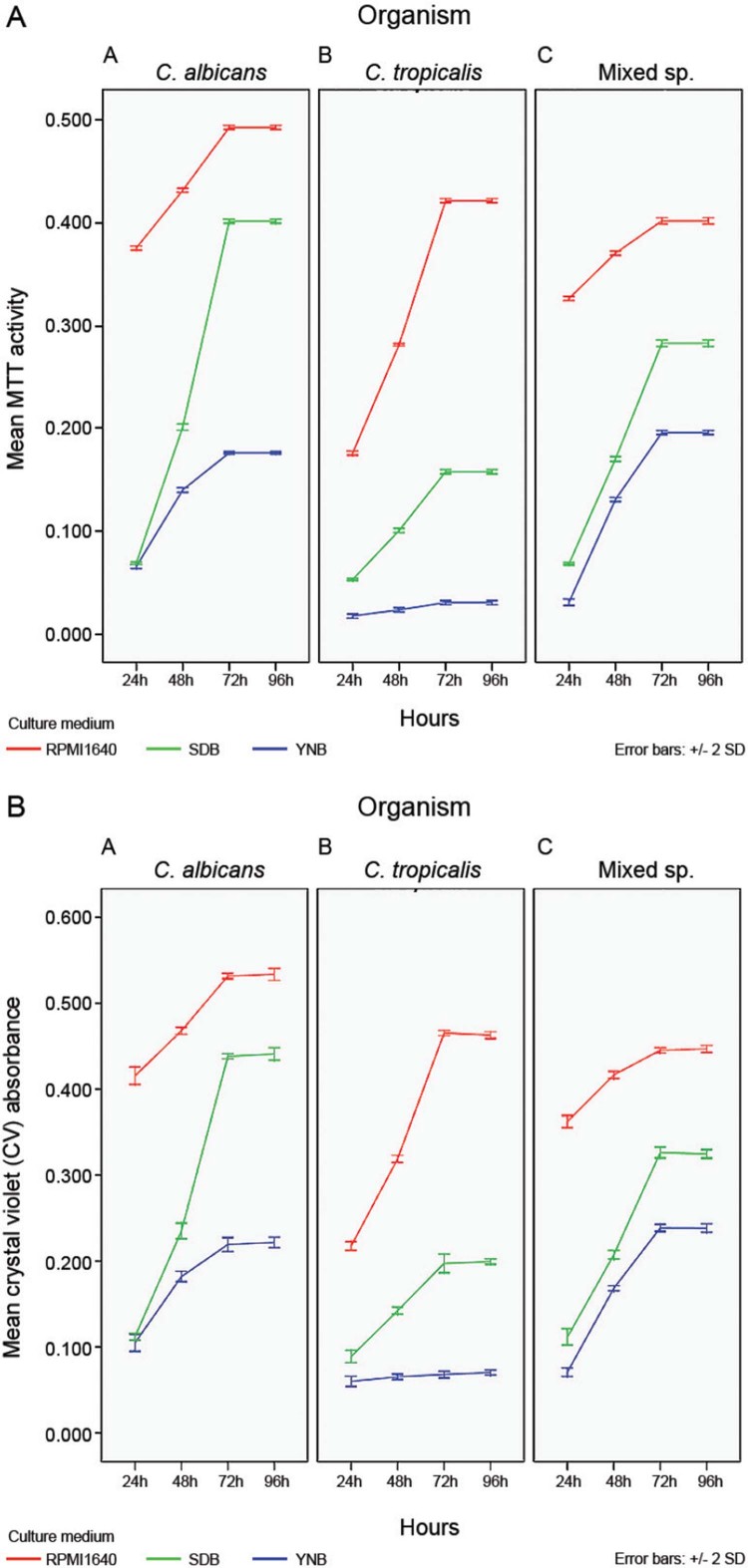
: relative biofilm formation of *Candida* species in three different culture media over a 96 h period. (A) MTT assay (B) crystal violet (CV) assay. Data are mean ± standard deviation of three independent experiments performed in triplicates.

*Biofilm growth* - Growth of biofilms were quantified using both CV and MTT assays. The results of CV and MTT assays correlated well (Fig. 3A-B). Growth of all three biofilms was uniformly high and reached a plateau at 72 h in all three culture media. The maximum growth of all three biofilms was noted with RPMI 1640 followed by SDB and YNB (Fig. 3A-B). The latter medium seemed to be least supportive of biofilm growth in general. Further, *C. tropicalis* biofilm growth in YNB was minimal compared with other media (Fig. 3A-B).

**Fig. 4:**
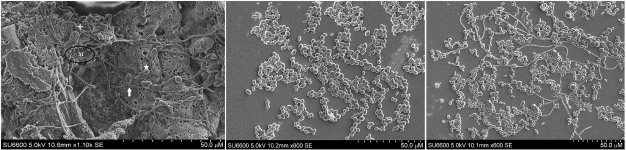
: (A) scanning electron micrograph of a 48 h old, *Candida albicans* biofilm in RPMI 1640 medium. Note the architecture of 72 h old mature biofilm with profuse extracellular matrix (M), hyphal elements (white solid arrow) blastopores (★), some bearing bud-scars (+) (Scale indicates 50.0 µM); (B) scanning electron micrograph of a 48 h old, *C. tropicalis* biofilms in RPMI 1640 medium. Note the architecture of 72 h old mature biofilm devoid of extracellular matrix and relatively sparse growth compared to Fig. 4A above (Scale indicates 50.0 µM); (C) scanning electron micrograph of a 48 h old, 1:1 mixed species biofilm of *C. albicans* and *C. tropicalis* in RPMI 1640 medium. Note the architecture of 72 h old mature of 1:1 mixed species biofilm in RPMI 1640 medium devoid of extracellular matrix but clearly showing hyphal elements of *C. albicans* intermixed with *C. tropicalis* blastospores devoid of hyphae (Scale indicates 50.0 µM).

*Ultrastructure of biofilms in RPMI 1640 medium* - In quantitative terms, as RPMI 1640 induced most biofilm growth in the two yeast species. Hence we further qualitatively evaluated their ultrastructure after 72 h growth. On SEM, marked hyphal development and a rather profuse extracellular matrix were noted with *C. albicans* in RPMI 1640 (Fig. 4A).

This was not the case for *C. tropicalis* biofilms, which was totally devoid of either hyphal elements or an extracellular matrix (Fig. 4B). On the other hand, in the dual species biofilms of *C. albicans* and *C. tropicalis*, a mixed picture was seen with moderate numbers of hyphal elements of *C. albicans* interspersed with blastospore aggregates of *C. tropicalis* (Fig. 4C). Overall these results generally support the data from the adhesion assay, where *C. albicans* biofilm growth enhancement was particularly profuse in RPMI 1640 medium.

## DISCUSSION

*C. albicans* is the commonest human oral fungal pathogen followed by *C. tropicalis.* Both species are found in nature as co-inhabitants in the oral cavity (Samaranayake et al. 1987). Morbidity or co-morbidity due to these species are extensive due to their potential to form biofilms on both biotic, as well as abiotic surfaces (e.g., venous catheters, implants) (Jin et al. 2004, Zijnge et al. 2010). Adhesion to latter surfaces is an essential prerequisite for subsequent biofilm development, and the biomass of a mature biofilm depends to a great extent on the degree of such initial adhesion. Hence in this investigation we monitored the effect of the three most commonly used culture media for *Candida*, on their planktonic growth, adhesion and biofilm formation.

In general, the planktonic growth, as opposed to biofilm growth, of the mono and the co-culture of the two yeast species was most profuse in SDB, followed by YNB and RPMI 1640. Both SDB and YNB media have a high content of glucose (20 g/L and 18 g/L, respectively) in comparison to RPMI 1640 medium (2 g/L glucose). The copious growth of *Candida* species in glucose-rich media and its clinical implications have been reported previously (Samaranayake & MacFarlane 1985, Samaranayake et al. 1986) and our data further confirm these findings.

As regards the yeast adhesion to the substrate, *C. tropicalis* exhibited the maximal adhesion in YNB medium supplemented with 100 mM glucose. On the other hand, *C. albicans* and the mixed species, co-culture exhibited maximal adhesion with RPMI 1640 medium, and lastly all three biofilm showed the maximal growth in RPMI 1640.

Compared to YNB and SD broth, RPMI 1640 is a nutrient-rich medium which contains amino acids (aas) and also known to mimic the composition of human fluids (Kucharíková et al. 2011). RPMI 1640 contains a variety of aas including high concentration of L-Glutamine, L-Arginine, L- Asparagine as well as vitamins and inorganic salts. However compared to YNB and SDB, glucose content is low in RPMI 1640 medium. This may be one reason for the higher planktonic growth noted in SDB which had the highest glucose concentration of the three growth media. RPMI 1640 is recommended for determining the susceptibility of planktonic cell for antifungals using MIC (micro and macro dilution methods) according to the NCCLS M27-A3 protocol (CLSI 2008).

On the other hand, aas are not constituents of either YNB or SD broth and they have a rich peptone and glucose content. This high glucose content may favor the planktonic growth of Candida. The presence of higher concentrations of readily available aas will support a favorable biofilm growth in RPMI medium compared to more complex peptone containing medium.

This medium has also been used previously for *C. albicans* attachment on polyurethane prior to subcutaneous catheter implantation in vivo (Kucharíková et al. 2010, 2011). As reported above this finding contrast with very high planktonic growth of *C. albicans*, *C. tropicalis* and their co-cultures with SDB but not with RPMI 1640. This observation is instructive, as it illustrates starkly the variations in substrate utilization by *Candida* during different growth phases.

Although *C. albicans* in vitro biofilm growth has been extensively studied there are limited studies on the biofilm forming ability of *C. tropicalis*. Interestingly we were unable to find any reports in the literature on neither the mixed biofilm growth of *C. albicans* and *C. tropicalis* nor the effect of media on biofilm formation of *C. tropicalis*.

Turning to the biofilm forming ability of the two species, clearly *C. albicans* was a far superior biofilm former than *C. tropicalis*, and RPMI 1640 nurtured the maximal biofilm growth relative to the other two media. These observations agree with the findings of Al-Fattani and Douglas (2006). Although *C. albicans* biofilm forming ability has been extensively studied there is no data on the mixed biofilms of these two species to compare with the current findings.

In quantitative terms, as RPMI 1640 induced most biofilm growth in the two yeast species, we further evaluated the qualitative nature of their ultrastructure. The architecture of biofilms in RPMI 1640 clearly confirmed the quantitative data. The complex organization of *C. albicans* biofilm with a dense growth of yeasts with ramifying hyphae, budding elements and bud scars with a profuse extracellular matrix were clearly evident in SEM micrographs. These results are in agreement with previous workers who reported RPMI 1640 heightened the hyphal formation in *C. albicans* biofilms (Kucharíková et al. 2011). In contrast, *C. tropicalis* biofilms in RPMI 1640 were devoid of hyphae although we noted increased blastospore density and micro colony development. Surprisingly, the latter biofilms also lacked an extracellular matrix even though it is known that biofilms of *C. tropicalis* also synthesized large amounts of extracellular polymeric material as well as hyphae (Bizerra et al. 2008). One reason for the conflicting observations could be the fresh clinical strains of *C. tropicalis* and the polyvinyl chloride (PVC) substrate used by previous workers (Bizerra et al. 2008). Additionally, inter-species and inter-strain heterogeneity of biofilm architecture could account for the differing observations between researchers. Finally, the mixed culture biofilms clearly depicted the disparate biofilm attributes of the two species with distinct blastospores of *C. tropicalis* devoid of hyphae interlaced with hyphae-bearing *C. albicans.* The mixed-biofilm growth however was relatively sparse and essentially largely devoid of an extracellular matrix. Hence we hypothesised a possible synergistic growth and biofilm forming effect when the two *Candida* species in co-culture. Yet, such a finding was not observed. In vitro, *C. albicans* co-aggregates and metabolically interacts and forms biofilms with a range of bacteria (Shirtliff et al. 2009, Thein et al. 2009). Studies of *Streptococcus mutans* and *C. albicans* biofilm co-culture have provided evidence for enhanced biofilm formation of both *S. mutans* and *C. albicans* (Falsetta et al. 2014, Sztajer et al. 2014). It is known that *C. albicans* promotes the biofilm formation of *S. mutans* in vitro. However based on our results of the two studied *Candida* species no such effect was noted in co-culture.

In conclusion, *C. albicans* and *C. tropicalis* displayed strikingly variable, heterogeneous growth, adhesion as well as biofilm forming potential and architecture in different growth media. From our data, we conclude that RPMI 1640 medium in general has the most potency to initiate and development of in vitro biofilms of the two tested *Candida* species. Considering the marked variations in *Candida* cell adhesion and biofilm development in different media described above, it is tempting to propose that RPMI 1640 should be considered by researchers in this field as a choice medium for such experiments in order to generate consistent and reliable data for cross comparison purposes. However, it is essential to evaluate a spectrum of other pathogenic *Candida* species in this medium before substantive conclusions are made.
