# ERRATUM: Culture media profoundly affect Candida albicans and Candida tropicalis growth, adhesion and biofilm development

**DOI:** 10.1590/0074-02760200294ER

**Published:** 2020-10-19

In the article **“Culture media profoundly affect *Candida albicans* and *Candida tropicalis* growth, adhesion and biofilm development”**, DOI number: 10.1590/0074-02760160294, published in *Mem Inst Oswaldo Cruz*, Rio de Janeiro, Vol. 111(11): 697-702, 2016:

On page 701, Fig. 4B-C should be replaced by the Fig. 4Bs-Cs below:

**Fig. 4:**
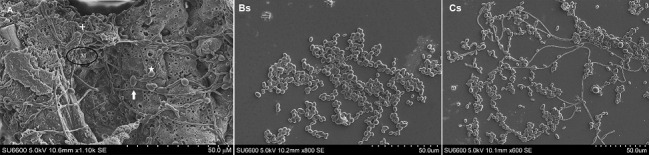
(A) scanning electron micrograph of a 72 h old, *Candida albicans* biofilm in RPMI 1640 medium. Note the architecture of 72 h old mature biofilm with profuse extracellular matrix (M), hyphal elements (white solid arrow) blastopores, some bearing bud-scars (+) (Scale indicates 50.0 μM); (Bs) scanning electron micrograph of a 72 h old, *C. tropicalis* biofilms in RPMI 1640 medium. Note the architecture of 72 h old mature biofilm devoid of extracellular matrix and relatively sparse growth compared to Fig. 4A above (Scale indicates 50.0 μM); (Cs) scan ning electron micrograph of a 48 h old, 1:1 mixed species biofilm of *C. albicans* and *C. tropicalis* in RPMI 1640 medium. Note the architecture of 72 h old mature of 1:1 mixed species biofilm in RPMI 1640 medium devoid of extracellular matrix but clearly showing hyphal elements of *C. albicans* intermixed with *C. tropicalis* blastospores devoid of hyphae (Scale indicates 50.0 μM).

